# A Rare Case Report of Extraosseous Ameloblastic Carcinoma and Review Article on Diagnosis and Treatment Dilemma

**DOI:** 10.1155/2024/4289276

**Published:** 2024-01-09

**Authors:** Sukkarn Themkumkwun, Puangwan Lapthanasupkul, Kanin Arunakul

**Affiliations:** ^1^Department of Oral and Maxillofacial Surgery, Faculty of Dentistry, Mahidol University, Bangkok, Thailand; ^2^Department of Oral and Maxillofacial Pathology, Faculty of Dentistry, Mahidol University, Bangkok, Thailand

## Abstract

Peripheral ameloblastic carcinoma is an extremely rare odontogenic carcinoma. Its histopathological feature is identical to the intraosseous type. This case report details a case of peripheral ameloblastic carcinoma at the right posterior maxilla region in a 60-year-old Thai male. The patient underwent a definitive treatment by partial maxillectomy and reconstruction with buccal fat pad. After 1-year follow-up, no recurrence of the lesion was found.

## 1. Introduction

Peripheral ameloblastoma (PA) is a rare peripheral odontogenic tumor [1]. The PA accounts for 2-10% of all ameloblastomas [2]. Malignant differentiation of ameloblastoma is very rare since only one peripheral ameloblastic carcinoma (PAC) was identified among 160 cases of the peripheral odontogenic tumors [2]. This peripheral tumor is differentiated from the intraosseous type by its location and bony involvement. Histologically, the PAC shares the same histological appearance as intraosseous ameloblastic carcinoma, consisting of ameloblastomatous islands with cellular atypia.

In this report, we present a detailed discussion of a rare case of PAC identified in a 60-year-old male patient at the posterior maxilla region. The paper provides comprehensive coverage of the clinical differential diagnosis, additional investigative measures implemented, as well as the treatment regimen administered to the patient for the management of PAC.

## 2. Case Presentation

**Figure 1:**
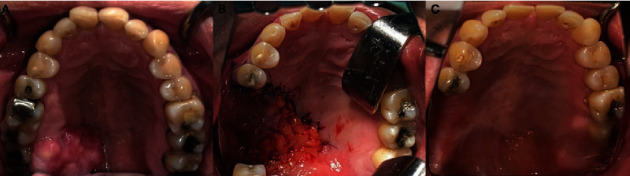
Intraoral examination revealed 3 × 2 cm, irregular-shaped firm mass at palatal gingiva of the right maxillary extending to the midpalate area (A). The maxilla after reconstruction with buccal fat pad and buccal flap advancement (B). The maxilla after 2-year follow-up showed complete mucosalization without scar contraction (C).

**Figure 2:**
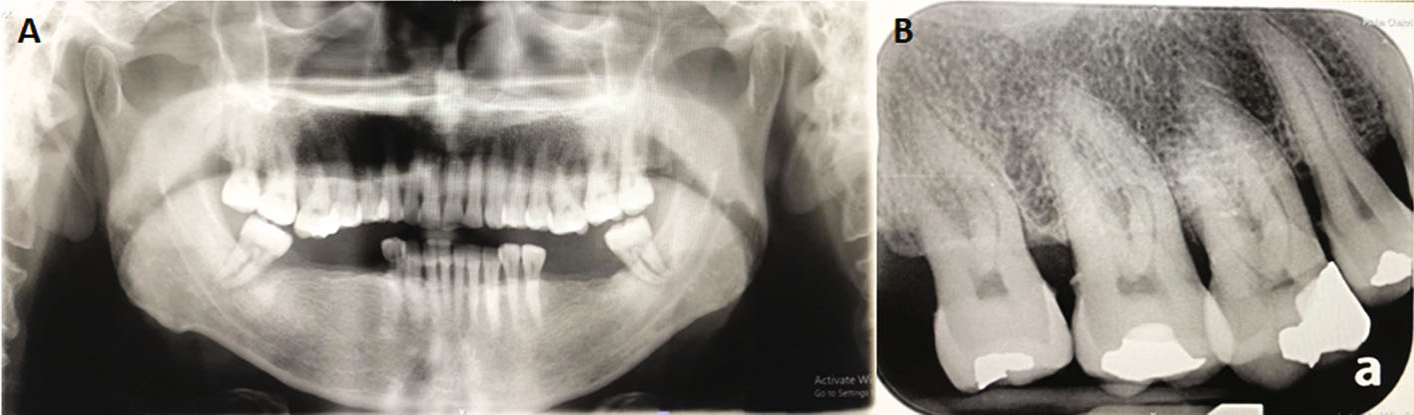
Panoramic radiograph shows no sign of intrabony tumor (A). Periapical radiograph shows generalized vertical bone resorption suspected due to chronic periodontitis and furcation involvement at right maxillary molar teeth (B).

A 60-year-old male came with a large mass at the right maxilla for more than a year. The patient denied any underlying diseases, drug allergy, and did not take any medication regularly. Tobacco and alcohol drinking were denied by this patient. Extraoral examination revealed normal facial form without cervical lymphadenopathy. Intraoral examination showed an irregular-shaped firm mass at the palatal gingiva of the right third maxillary molar extending to the midpalate area, sized 3 × 2 cm (Figure 1(A)). No tenderness and paresthesia were found. Panoramic and periapical radiographs show vertical bone loss with furcation involvement at the right maxillary molar teeth, suspected as a result of periodontitis (Figures 2(A) and 2(B)).

**Figure 3:**
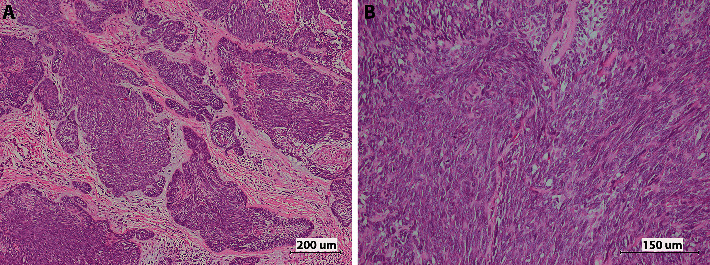
Histopathologic features show irregular-shaped islands of malignant ameloblastic epithelial cells with cellular crowding (A). Malignant spindle cells arrange in streaming fascicles whereas a few stellate reticulum-like cells are observed in small foci. The tumor cells demonstrate cytological atypia (B).

Due to the COVID-19 pandemic at that time causing a limitation of dental operation visits, an excisional biopsy was considered even though the character of the lesion is inconspicuous. Histopathological examination reveals a mucosal mass fully infiltrated by malignant epithelial cells. The malignant epithelial cells are forming islands and anastomosing cords. Several tumor islands show spindles cells with remarkable cellular atypia in the center while squamous differentiation or stellate reticulum-like cells are recognized in a few areas. A few tumor nests with the normal architecture of ameloblastoma are detected. The histopathological features were consistent with peripheral ameloblastic carcinoma with the positive margins (Figures 3(A) and 3(B)).

**Figure 4:**
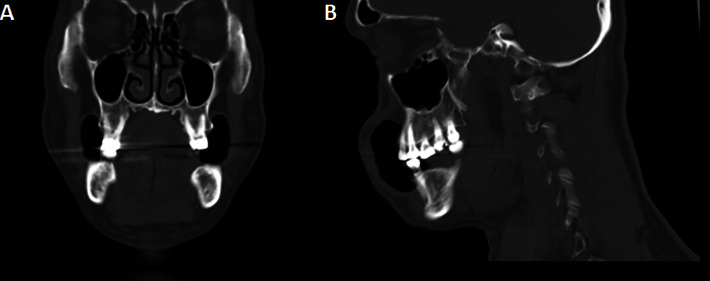
Coronal section of bone window medical computed tomography image shows no sign of bony invasion (A). Sagittal section of bone window medical computed tomography image shows no sign of bony invasion (B).

One month after the excision, the palatal gingiva at right maxillary third molar area still had redness and tenderness. After discussing the risks and benefits of further surgery, the patient decided to undergo further surgical resection. Routine malignancy workups were subsequently performed, including medical computed tomography (CT) with contrast of the brain, neck, chest, and whole abdomen. Only subcentimeter cervical lymph nodes were presented at cervical levels IB, IIA, III, IV, and V bilaterally. Physical examination showed normal findings, and there is no specific symptom observed in other organs. Based on these findings, we summarize that no metastasis was detected. The subcentimeter lymph nodes were suspected as a reaction after surgical treatment. No obvious bony destruction is detected (Figures 4(A) and 4(B)). Sulcular incision was extended from the right maxillary first molar to the right tuberosity. The wide excision at the palatal mucosa was performed along with one-centimeter margin with bony resection at the alveolar bone of the right first maxillary molar to the tuberosity. Before reconstruction of the defect, frozen sections of all mucosal margins were taken and examined. The results of the frozen section analysis indicated the absence of tumor cells in any of the examined margins. Then, the maxillary defect was reconstructed with buccal flap advancement and buccal fat pad (Figure 1(B)).

The patient was admitted to the general ward for five days postoperatively. The symptoms kept getting better during admission and the patient was discharged. Nasogastric tube was inserted, and palatal stent was retained for 2 weeks. One year postoperatively, the wound completely healed, and neither oronasal fistula nor sign of recurrence was observed. The final pathological examination shows no tumor cell in the bony part of the specimen. Accordingly, peripheral ameloblastic carcinoma was diagnosed. A two-year follow-up showed no signs of recurrence (Figure 1(C)). Medical CT scans were performed annually to rule out any recurrence or metastasis of the tumor. The scans included the brain, chest, neck, and abdomen. The results indicated that all the organs were within normal limits, suggesting no sign of recurrent or metastatic tumor.

## 3. Discussion

Ameloblastoma is a common benign odontogenic tumor in the maxillofacial region. The malignant counterpart of ameloblastoma is extremely rare and categorized into metastasizing ameloblastoma and ameloblastic carcinoma. According to the World Health Organization Classification of Tumors in 2017, ameloblastic carcinoma combines the histological features of ameloblastoma and cytological atypia [3]. Peripheral ameloblastic carcinoma (PAC) was not mentioned due to its rarity. Only 8 cases of PAC were published in English literature since 1982 as shown in Table 1 [4–10].

The age of the PAC patients ranged from 47 to 85 years old with a mean of 75 years old. The high frequency in the elderly may suggest the earlier lesions of PA and can progress to the malignant form [11]. Our case was 60 years old, corresponding to that age group. The clinical presentation of most PAC is a firm mass with an irregular surface as presented in our case. Most of the lesions presented without pain or bleeding [4, 8, 9]. One patient had an uncomfortable feeling due to the large size of the lesion [12]. Pain was reported probably resulting from the mass-interfering occlusion [6]. As the diagnosis of the peripheral lesion, the primary location of the lesion in all reported cases was at the gingiva and the bone involvement was presented only with a large tumor mass invading the bone underneath. Generally, bone involvement was not found except for superficial bone resorption due to tumor pressure [10]. In contrast, the radiographic feature showing either radiolucent or distinct osteolytic lesion within the bone usually indicates the intraosseous ameloblastic carcinoma.

Clinically, the differential diagnosis of peripheral ameloblastic carcinoma includes the peripheral types of other odontogenic tumors such as peripheral odontogenic myxoma and peripheral odontogenic fibroma [13]. Peripheral odontogenic fibroma is the most common peripheral epithelial odontogenic tumor and accounted for 51.1%, followed by peripheral ameloblastoma and peripheral calcifying cystic odontogenic tumor, respectively [5]. However, the more common lesion is reactive or inflammatory lesions such as peripheral giant cell granuloma, peripheral ossifying fibroma, and pyogenic granuloma. The differences between the reactive lesions and the peripheral tumors include onset, suspected causative factors, and location of the lesion. The reactive lesions frequently present with an obvious cause, such as continuous trauma or an increasing of hormones during pregnancy, while the peripheral tumors develop without a cause. The onset of the reactive lesion usually started with the beginning of cause while the peripheral tumor could arise without an exact starting point. The location of the reactive lesion was commonly found at the gingiva, followed by the vestibule, the buccal mucosa, and the tongue, respectively. The location of the lesion depends on the irritating cause. In contrast, the peripheral odontogenic tumor is found only at the alveolar gingiva [5]. In our presented case, the lesion was found at the alveolar palatal gingiva of the right maxillary third molar extending to the midpalate area. This is not a common location of the reactive lesion since no suspected irritative factor was noted.

Due to the malignancy in nature of PAC, further investigation is one of the significant steps prior to surgery. Chest radiograph should be done before surgical planning to rule out a metastatic tumor. CT is necessary for planning bony surgical margin in the case of questionable plain film radiographs or suspected bony invasion from clinical appearance. Additionally, CT has a role in evaluating cortical bone resorption. Magnetic resonance imaging (MRI) can early detect bone marrow invasion but not a routinely used method. A previous report shared a case of PAC at the lingual gingiva of the right second mandibular premolar without bony invasion in plain film and CT, but MRI showed a high-signal area on the T2-weighted image [12]. For our case, only CT was done and no evidence of obvious bony invasion. Magnetic resonance imaging (MRI) was not performed in this case because there was no evidence of tumor invasion into the palate and maxillary bone. From the surgeon's perspective, the bone may act as a barrier to prevent tumor invasion from the oral mucosa to the base of the skull. As long as there is no presence of tumor cells within the bone, invasion into the skull base is not likely to be present. Additionally, it should be noted that MRI availability may be limited in certain local hospital settings and the cost is higher, making it less accessible in our particular case. However, some parts of the palatal bone were resected for oncological safety purposes.

Since PAC is a very rare disease, the standard surgical treatment in PAC has not been yet defined. Buchner et al. suggested a wide tumor excision with underlying bone removal [6] whereas Sumita et al. suggested one-centimeter margin of hard and soft tissue in early phase [12]. The resection margin recommended was less than that recommended for the intrabony ameloblastic carcinoma due to a low extension of the lesion [12]. However, the exact surgical margin has not been summarized due to its rarity. Moreover, the behavior of peripheral odontogenic carcinomas is less aggressive than that of the intraosseous carcinoma [12]. The surgical margin of 2-3 cm is thus not recommended in the peripheral type [12]. The exact surgical excision margin should start with one centimeter with further details decided by the attending surgeon individually, and large series of cases would provide further recommendations. However, local excision is not recommended since two of the reported studies had a recurrence after local excision [4, 8]. In addition, rapid surgical treatments within one month after diagnosis were suggested to reduce the risk of progression [12]. Our patient underwent partial maxillectomy three months after the first operation. However, spreading of the lesion to other sites or expanding over the existing wound was not reported. The role of chemotherapy and radiotherapy was not generally mentioned. Lin et al. reported that PAC is resistant to chemotherapy and radiotherapy [8] whereas Baden et al. presented a case in which adjunctive radiotherapy may play role in the progressive dedifferentiation of ameloblastic carcinoma to anaplastic carcinoma [4].

In other head and neck malignancies, particularly squamous cell carcinoma, the occurrence of neck metastasis is relatively high. Prophylactic neck dissection is commonly accepted as a standard practice. However, the standard for neck dissection in negative neck nodes in PAC has not been established. In this case, routine neck dissection was not performed. Cervical neck dissection is recommended if the size of lymph nodes exceeds 1 cm in levels II, III, and IV or exceeds 1.5 cm in submandibular lymph nodes, as well as when irregular shape or central necrosis is observed. Since the subcentimeter node observed in our case does not meet these criteria, we plan to observe and follow up annually using medical CT with contrast.

A routine follow-up period should be considered in PAC. Some evidence showed local recurrence to distant metastasis [4, 8]. Wettan, et al. reported a case of a 71-year-old man who underwent surgical excision of peripheral ameloblastoma at the mandibular gingiva. Approximately one year postoperatively, the patient returned with a whitish lesion at the previous biopsy area, pathologically diagnosed as severe dysplasia [14]. Despite the nonaggressive behavior of PAC, a long-term follow-up is still necessary due to various fashions of recurrence. The follow-up should also include clinical examinations and medical CT with contrast for surveillance of metastasis.

## Figures and Tables

**Table 1:** Reported cases of peripheral ameloblastic carcinoma cases.

Author (year)	Gender	Age (years)	Location	Symptoms and clinical presentations	Radiographic examination	Treatment	Follow-up
Lin (1987) [8]	Female	83	Right mandibular alveolus	Tumor mass without pain	Concave depression of bone below area of swelling	Local excision	Present of supraclavicular lymph node at 2 days postoperative and die at 7 days postoperative due to pulmonary complications
McClatchey et al. (1989) [9]	Female	77	Left maxillary tuberosity	Tumor mass	NA	Local excision	No recurrence at 24 months postoperative
E. Baden et al. (1993) [4]	Male	82	Left maxillary tuberosity	Tumor mass without pain	Saucerization of tuberosity	Local excision	2 times of recurrence within 6-year follow-up period
Califano et al. (1996) [6]	Male	47	Left maxillary canine region	Painful tumor mass with fixation to surrounding tissue	No evidence of bony invasion	Local excision of soft tissue with excision of bone surround lesion	No recurrence at 12 months postoperative
Buchner et al. (2006) [5]	The detail of the patient is unavailable						
Saito et al. (2016) [10]	Male	82	Right palatal gingiva	Hemorrhagic mass without pain	Evidence of bone resorption in CT and MRI	Tumor resection with margin 5 mm	Died at 21 months after surgery from multiorgan failure
Sumita et al. (2020) [12]	Female	85	Left mandibular alveolus	Uncomfortable feeling	Evidence of bone resorption in CT and diffuse high-signal area in MRI	Mandibular resection with margin 2 cm	No recurrence at 9 years postoperative
Sumita et al. (2020) [12]	Male	71	Right mandibular premolar region	Gingival swelling with persistent bleeding	No evidence of bone resorption in CT but tumor mass was identified in MRI	Mandibular resection with margin 2 cm	No recurrence at 9 years postoperative

(CT = computed tomography; MRI = magnetic resonance imaging; NA = the detail of patient is unavailable).

## Data Availability

To obtain the information presented in this study, please contact the corresponding author for assistance, as the data cannot be accessed publicly due to ethical considerations.
